# A bushel of viruses: Identification of seventeen novel putative viruses by RNA-seq in six apple trees

**DOI:** 10.1371/journal.pone.0227669

**Published:** 2020-01-13

**Authors:** Alice A. Wright, Alex R. Cross, Scott J. Harper

**Affiliations:** Department of Plant Pathology, Washington State University, Prosser, WA, United States of America; Oklahoma State University, UNITED STATES

## Abstract

Apple decline in Washington state has been increasing in incidence, particularly on Honeycrisp trees grown on G.935 rootstock. In this disease the trees exhibit dieback with necrosis at the graft union and in the rootstock. The cause of this disease remains unknown. To identify viral candidates, RNA-seq was performed on six trees: four trees exhibiting decline and two healthy trees. Across the samples, eight known viruses and Apple hammerhead viroid were detected, however none appear to be specifically associated with the disease. A BLASTx analysis of the RNA-seq data was performed to identify novel viruses that might be associated with apple decline. Seventeen novel putative viruses were detected, including an ilarvirus, two tombus-like viruses, a barna-like virus, a picorna-like virus, three ourmia-like viruses, three partiti-like viruses, and two narna-like viruses. Four additional viruses could not be classified. Three of the viruses appeared to be missing key genes, suggesting they may be dependent upon helper viruses for their function. Others showed a specific tropism, being detected only in the roots or only in the leaves. While, like the known apple viruses, none were consistently associated with diseased trees, it is possible these viruses may have a synergistic effect when co-infecting that could contribute to disease. Or the presence of these viruses may weaken the trees for some other factor that ultimately causes decline. Additional research will be needed to determine how these novel viruses contribute to apple decline.

## Introduction

Apple decline, in which the afflicted trees exhibit dieback along the branches and eventual death, has been observed in certain scion/rootstock combinations, including Honeycrisp and Honeycrisp variants on G.935 rootstock, in Washington state. A similar disease is affecting apple trees in the eastern United States and Canada, specifically Fuji, Gala, Golden Delicious, and Crimson Crisp on M9 rootstock and Gala on M26 rootstock [1–3]. In the eastern US, the affected trees exhibit symptoms of dieback on the branches, often with the presence of cankers at the graft union and necrosis moving up the graft union into the scion tissue [1, 3]. Interestingly, the rootstock appears to be healthy, sending out suckers while the scion material is dying back [1, 3]. In contrast, in declining Honeycrisp/G.935 trees in Washington state, necrosis is observed at the graft union and in the rootstock stem and root tissues. No rootstock suckers are observed and the root systems are much smaller than healthy trees grown in the same block (sometimes in the next row). What is common between decline in Washington and in the eastern United States and Canada is that the declining trees are often scattered among healthy trees [1, 3]. Identifying a cause or causes of this disease has been difficult. In Pennsylvania, Apple luteovirus 1 (ALV1) has been observed in some, but not all, declining plants, as were Apple chlorotic leafspot virus (ACLSV), Apple stem grooving virus (ASGV), and Apple stem pitting virus (ASPV) [2]. These viruses were also observed in asymptomatic trees, making it unlikely that they are the sole cause of this disease [2]. A recent study of declining trees in the eastern United States examined weather conditions, soil conditions, common viruses, and the microbial rhizosphere population [4]. No differences were observed between symptomatic and asymptomatic plants, excepting a decrease in one bacterial population in the rhizosphere of declining trees that may have been indicative of water stress [4].

Next generation sequencing (NGS) has enabled researchers in the last few years to assess the virome of a plant [5]. This technology has been applied to several crop species including peaches [6], lilies [7, 8], barley [9], sweet pepper [10], grapevine [11, 12], vanilla [13], and citrus [14]. While these studies detected known species, novel viruses were also identified [7, 10, 13, 14]. NGS is particularly useful when investigating diseases where the pathogen has yet to be identified. Recently, NGS has been used to identify two viruses associated with apple rubbery wood disease [15] and one associated with citrus concave gum disease [16]. ALV1 was also discovered by NGS [2].

In an effort to identify a virus associated with apple decline in Washington state, RNA-seq was performed on symptomatic and asymptomatic trees. In the process, several new viruses were discovered, illustrating the difficulty in associating the presence of viruses with a given disease.

## Materials and methods

### Plant material

Plant tissue (leaves and roots) from one to two year old whole apple trees exhibiting symptoms of apple decline were collected from commercial orchards in eastern Washington with the permission of the owners (Table 1). For comparison, tissue from a healthy G.935 rootstock maintained at the Clean Plant Center Northwest in Prosser, WA was also collected.

**Table 1:** Trees sequenced in this study.

Tree	Scion/Rootstock	Disease Status	Location	Tissue Sequenced
1	Firestorm/G935	Symptomatic	Central Washington	Leaf and root
2	Firestorm/G935	Symptomatic	Central Washington	Leaf and root
3	Firestorm/G935	Symptomatic	Central Washington	Leaf and root
4	G935	Healthy	Prosser, Washington	Leaf only
5	Sugar Bee/M9	Healthy	Northern WA	Root only
6	Honeycrisp/G935	Symptomatic	Central Washington	Root only

### RNA extractions and sequencing

For each collected sample, RNA was extracted using the RNEasy plant mini kit (Qiagen Corp., Germantown, MD). Briefly, ~100 mg of root or leaf tissue was frozen at -80°C for 1 hr, then homogenized by pulverization in a Tissuelyzer bead-beater (Qiagen). Total RNA was extracted as previously described [17]. The Qubit RNA broad range and RNA IQ assays (Fisher Scientific, Hanover Park, IL) were used to assess RNA quantity and quality. Only those samples that met the criteria for sequencing were submitted to Genewiz (South Plainfield, NJ). All samples were DNAse treated, ribosomal RNA depleted with the Ribo-Zero Gold kit, and libraries constructed using an Illumina TruSeq Stranded Total RNA Library Prep Kit. Paired-end 150 bp libraries were prepared and sequenced on one lane of an Illumina HiSeq platform.

### Data analysis

Raw sequencing reads were analyzed using CLC Genomics Workbench v12 (Qiagen). Reads were trimmed to remove adaptors, then mapped to the apple genome (accession number GCA_002114115.1) [18] to eliminate host sequences, and unmapped reads collected. The non-host reads were used for de novo assembly for each sample. All assemblies had a word size of 20 and a minimum contig length of 200 bp. BLAST analysis was performed on each contig using the viral, viroid, and nucleotide databases (ref_viruses_rep_genomes, ref_viroids_rep_genomes, and nt; downloaded into CLC Genomic Workbench November 2018). Contigs without BLASTn matches were subjected to BLASTx analysis. Raw reads were submitted to GenBank under accession number PRJNA562540. Accession numbers for the assembled viral contigs are provided in S1 Table.

For known viruses found in the NGS samples, trimmed reads were assembled to the viral genome for each sample. This was done in CLC Genomics Workbench using default settings. Reads mapped, percent of total reads per sample, and percent coverage of the known viral genome were recorded. Accession numbers of viral genomes are provided in S2 Table.

Primers were designed based on sequence assemblies to detect novel putative viral sequences by RT-PCR. Reaction conditions consisted of ~50 ng RNA, 400 nM forward and reverse primers, 1X reaction mix, 0.2 μL ssiii/taq and water to 10 μL (Invitrogen^TM^ Platinum^TM^ Superscript^TM^ III One Step RT-PCR System, Fisher Scientific). Primer sequences and cycle conditions for each virus are in S3 Table. PCR products were analyzed on a 1% agarose 1X TAE gel stained with Sybr Safe (Fisher Scientific).

Additional apple samples not submitted for sequencing were screened for the presence of known apple viruses. RT-PCR assays were performed, as described above, for ARWaV-1 and ARWaV-2 [15], AhVd [19], and CCGaV [16]. qRT-PCR was performed for AGCaV and ASGV [17] and ACLSV, ApMV, and ASPV [Harper, manuscript in review] using the Invitrogen^TM^ Superscript^TM^ III Platinum^TM^ One-Step qRT-PCR kit (Fisher Scientific).

### Phylogenetic analysis

Phylogenetic trees were constructed using MEGA-X [20]. The neighbor-joining method was used for protein sequences, using the Jones-Taylor-Thornton models [21, 22]. Bootstrapping was used with a total of 500 replications. Branches with bootstrap values of less than 50 were not considered significant and were collapsed. Bootstrap values greater than 70 were considered strong whereas those between 50 and 70 were regarded as weak [23].

## Results

### NGS

Illumina sequencing generated on average 75,500,000 reads per sample (Table 2). Of these, between 5 to 15 million reads per sample did not map to the apple genome, most likely being viral, bacterial and fungal in origin. De novo assembly was performed on unmapped reads for each sample. Leaf tissue had the fewest number of assembled contigs, ranging from 12,000 to 27,000. In contrast, in root tissue, there was a range of 124,000 to 449,000 contigs per sample, an approximate tenfold increase compared to leaf tissue.

**Table 2:** Number of reads and contigs obtained from sequencing and bioinformatics analysis.

Sample^a^	Trimmed Reads	Unmapped Reads^b^	Contigs
1L	77640779	6890692	12168
2L	98802984	8136476	27752
3L	60170411	5601674	20642
4L	73225264	5935916	23202
1R	76131744	15746746	276231
2R	86741788	15594634	298283
3R	69383205	22336836	449714
5R	61413909	7417204	124569
6R	75870421	12922122	212501

a. L indicates a leaf sample and R a root sample.

b. Reads that did not map to the apple genome.

### Known apple viruses

Several viruses known to infect apple trees were detected by NGS analysis (Table 3, in bold). All were positive for Apple mosaic virus (ApMV) and ASPV. The leaf tissue for tree 1 came back negative for Apple hammerhead viroid (AhVd) but the root tissue was positive; all other samples were positive for this viroid. All except the healthy G.935 rootstock (4L) were positive for ACLSV. Apple green crinkle associated virus (AGCaV) was detected in trees 1, 2, 5, and 6 and ASGV in trees 1, 5, and 6. The newly reported phleboviruses [15, 16] were also present in some of the samples with Apple rubbery wood associated virus 1 (ARWaV1) detected in tree 5, Apple rubbery wood associated virus 2 (ARWaV2) in trees 1, 3, and 6, and Citrus concave gum associated virus (CCGaV) in tree 6. An additional 48 trees were screened by qRT-PCR for the presence of these viruses. Including the sequenced trees, 15 trees were asymptomatic for decline and 38 were symptomatic. Among the trees, ARWaV1 and AGCaV were the least common, occurring in 4% and 9% of the samples, respectively. ASGV was most common, occurring in 74% of the samples, followed by ApMV and ASPV, which were both present in 64% of the samples. However, in reviewing the data, there are no clear trends between asymptomatic and symptomatic plants. No virus or combination of viruses appears to be associated with disease.

**Table 3:** Summary of known viruses present in trees symptomatic and asymptomatic for apple decline^a^.

Sample	Cultivar/Rootstock^b^	Tissue	Symptomatic	ACLSV	AGCaV	ApMV	ARWaV1	ARWaV2	ASGV	ASPV	CCGaV	AhVd
**1L**	**Firestorm/G.935**	**Leaf**	**Yes**	**+**	**+**	**+**	**-**	**-**	**+**	**+**	**-**	**-**
**1R**	**Firestorm/G.935**	**Root**	**Yes**	**+**	**+**	**+**	**-**	**+**	**+**	**+**	**-**	**+**
**2L**	**Firestorm/G.935**	**Leaf**	**Yes**	**+**	**+**	**+**	**-**	**-**	**-**	**+**	**-**	**+**
**2R**	**Firestorm/G.935**	**Root**	**Yes**	+	+	+	-	-	-	+	-	+
**3L**	**Firestorm/G.935**	**Leaf**	**Yes**	+	-	+	-	+	-	+	-	+
**3R**	**Firestorm/G.935**	**Root**	**Yes**	+	-	+	-	+	-	+	-	+
**4L**	**G.935**	**Leaf**	**No**	-	-	+	-	-	-	-	-	+
4R	G.935	Root	No	-	-	-	-	-	-	-	-	-
**6R**	**Honeycrisp/G.935**	**Root**	**Yes**	**+**	**+**	**+**	**-**	**+**	**+**	**+**	**+**	**+**
7L	G.41	Leaf	No	-	-	-	-	-	+	+	-	-
7S	G.41	Stem	No	-	-	-	-	-	+	-	-	+
8L	G.935	Leaf	No	-	-	-	-	-	+	+	-	-
8S	G.935	Stem	No	-	-	-	+	-	-	+	-	+
9L	Honeycrisp/G.935	Leaf	No	+	-	+	-	+	+	+	+	-
10L	Honeycrisp/G.935	Leaf	No	-	-	+	-	-	+	-	+	+
11L	Honeycrisp/G.935	Leaf	No	-	-	+	-	-	+	-	-	+
12L	Honeycrisp/G.935	Leaf	No	-	-	+	-	-	+	-	-	-
13L	Honeycrisp/G.935	Leaf	No	-	-	+	-	-	+	-	-	-
14R	Honeycrisp/G.935	Root	No	-	-	+	-	-	+	-	-	+
15LS	HoneycrispV1/G.935	Leaf/Stem	No	-	-	-	-	-	-	+	-	-
16LS	HoneycrispV1/G.935	Leaf/Stem	No	-	-	-	-	-	+	+	+	-
17R	HoneycrispV1/G.935	Root	No	-	-	-	-	-	+	+	+	-
17S	HoneycrispV1/G.935	Stem	No	-	-	-	-	-	-	+	-	+
18L	HoneycrispV1/P2	Leaf	No	-	-	+	-	-	+	+	+	-
19L	HoneycrispV1/P2	Leaf	No	+	-	-	-	-	+	+	+	-
20L	HoneycrispV1/P2	Leaf	No	+	-	+	-	+	+	+	+	-
21L	Honeycrisp	Leaf	Yes	+	-	-	-	+	+	+	+	-
22L	Honeycrisp	Leaf	Yes	+	-	-	-	+	+	+	+	-
23L	Honeycrisp	Leaf	Yes	-	-	-	-	-	+	-	-	-
24L	Honeycrisp	Leaf	Yes	+	+	-	-	+	+	+	+	-
25L	Honeycrisp/G.935	Leaf	Yes	-	-	+	-	-	+	-	-	-
26L	Honeycrisp/G.935	Leaf	Yes	+	-	+	-	+	+	+	-	+
27L	Honeycrisp/G.935	Leaf	Yes	+	-	+	-	+	+	+	-	+
28L	Honeycrisp/G.935	Leaf	Yes	+	-	+	-	+	-	+	-	+
29L	Honeycrisp/G.935	Leaf	Yes	-	-	+	-	-	-	-	+	+
30L	Honeycrisp/G.935	Leaf	Yes	+	-	+	-	-	-	+	-	+
31L	Honeycrisp/G.935	Leaf	Yes	-	-	+	-	-	-	-	-	+
32L	Honeycrisp/G.935	Leaf	Yes	-	-	+	-	-	+	-	-	-
33L	Honeycrisp/G.935	Leaf	Yes	+	-	+	-	-	+	+	-	+
34L	Honeycrisp/G.935	Leaf	Yes	-	-	+	-	-	+	-	-	+
35L	Honeycrisp/G.935	Leaf	Yes	+	-	+	-	+	+	+	+	-
36L	Honeycrisp/G.935	Leaf	Yes	+	-	+	-	+	+	+	-	-
37L	Honeycrisp/G.935	Leaf	Yes	+	-	+	-	+	+	+	+	-
38L	Honeycrisp/G.935	Leaf	Yes	+	-	+	-	+	+	+	-	+
39R	Honeycrisp/G.935	Root	Yes	-	-	+	-	+	-	-	-	+
40R	Honeycrisp/G.935	Root	Yes	-	-	+	-	+	-	-	-	+
41L	HoneycrispV1/G.935	Leaf	Yes	-	-	+	-	+	+	+	-	-
42L	HoneycrispV1/G.935	Leaf	Yes	-	-	+	-	+	+	+	+	-
43L	HoneycrispV1/G.935	Leaf	Yes	-	-	-	-	+	+	+	+	-
44L	HoneycrispV1/G.935	Leaf	Yes	-	+	+	-	+	+	+	+	+
45L	HoneycrispV1/G.935	Leaf	Yes	-	-	-	-	-	+	-	-	-
46L	HoneycrispV1/G.935	Leaf	Yes	-	-	-	-	-	+	+	-	-
47L	HoneycrispV1/G.935	Leaf	Yes	-	-	-	-	-	+	+	-	-
48L	HoneycrispV1/G.935	Leaf	Yes	-	-	-	-	-	+	+	-	-
49R	HoneycrispV1/G.935	Root	Yes	-	-	+	+	+	+	+	-	+
50L	HoneycrispV1/Nic29	Leaf	Yes	+	-	+	-	+	+	+	-	-
51L	HoneycrispV2	Leaf	Yes	+	-	-	-	-	-	-	+	-
52L	HoneycrispV2	Leaf	Yes	-	-	-	-	-	-	-	+	-
53L	HoneycrispV2	Leaf	Yes	+	-	-	-	-	-	-	-	-

a. Samples in bold are those for which next generation sequencing was performed. The remainder were confirmed by qRT-PCR and RT-PCR.

b. Two Honeycrisp variants, in addition to Firestorm, were included in this study.

The known viruses detected in each NGS sample were explored further by mapping back the trimmed reads to each virus for each sample. Number of reads mapped, percent of total reads mapped, and percent coverage of the viral genome were recorded for each virus for every sample in which it was detected (S2 Table). Some viruses were present in great abundance, specifically ACLSV, AGCaV, ASGV, and ASPV as well as the viroid AhVd, where reads assembled ranged from 0.01% to 0.69% of the total reads sequenced. The viruses with multipartite genomes, ApMV, ARWaV1, and ARWaV2, tended to be less abundant. An exception was CCGaV in sample 6R in which RNA1 and RNA2 were represented by 0.02% and 0.07% of the total reads, respectively. While some of the viruses were strikingly abundant, 0.61% of all reads were ASPV for sample 2R, there is no observable trend with regards to abundance of individual viruses and disease state. This is not unexpected because, as shown in Table 3, no one specific known virus or combination of viruses appears to be associated with disease.

### Novel apple viruses

Based on the screening of a larger number of apple samples (Table 3) there has been no clear link between any known viruses and apple decline. Therefore, to determine if a novel virus was present in the apple decline samples, BLASTx analysis was performed on contigs for which BLASTn (megablast) had failed to produce a match. Across all six trees, seventeen putative novel virus-like sequences were identified (Table 4). Each one of the contigs matched a known viral amino acid sequence, however the percent coverage and amino acid identity (25% to 61%) indicate that these are distinct and novel viruses. Some had limited homology to known plant viruses, such as Tobacco streak virus and Cowpea tombusvirid 1, while others had homology to fungal or insect viruses. Fourteen of the new viruses were detected in the root samples, while the remaining three were detected in leaf samples (Table 4).

**Table 4:** Putative novel viruses detected in apple samples.

Name^a^	Samples	Sequence Coverage	Contig length	BLASTx results	% Coverage	% Identity	E value
Apple barna-like virus 1	4L	40.78	4099	Riboviria sp RdRp (QDH90348)	25.00%	49%	4.00E-94
Apple ilarvirus 1 RNA2	5R	10.71	1058	Blackberry chlorotic ringspot virus replicase P2a (ARS65724.1)	99.90%	39%	1.00E-57
Apple ilarvirus 1 RNA3	5R	91.49	2124	Parietaria mottle virus movement protein (CAJ58667.1)	36.40%	44%	1.00E-67
Apple narna-like virus 1	3R	12.26	2511	Wenzhou narna-like virus 1 RdRp (APG77283.1)	71.60%	31%	1.00E-54
Apple narna-like virus 2	3R	29.05	2668	Wenzhou narna-like virus 1 RdRp (APG77283.1)	73.90%	26%	5.00E-36
Apple ourmia-like virus 1	3L	42.71	1856	Pyricularia oryzae ourmia-like virus 2 RdRp (BBF90577.1)	56.20%	50%	6.00E-96
Apple ourmia-like virus 2	5R	15.56	2570	Phomopsis longicolla RNA virus 1 RdRp (YP_009345044.1)	73.40%	53%	0
Apple ourmia-like virus 3	3R, 6R	93.05	3067	Cladosporium cladosporioides ourmia-like virus 1 RdRp (QDB74999)	55.00%	33%	4.00E-73
Apple partiti-like virus 1	1R, 2R,3R	234.3	2010	Partitiviridae sp. RdRp (QDH87388)	33.00%	81%	7.00E-128
Apple partiti-like virus 2	1R, 2R, 3R	374.44	1825	Partitiviridae sp. RdRp (QDH87090)	60.00%	40%	5.00E-79
Apple partiti-like virus 3	2R, 3R	783.33	1930	Partitiviridae sp. RdRp (QDH87090)	60.00%	35%	1.00E-51
Apple picorna-like virus 1	4L	39.05	11885	Polycipiviridae sp. RdRp (AZL87720.1)	29%	34%	1e-141, 2e-34
Apple tombus-like virus 1	3R, 5R	36.81	2971	Sanxia tombus-like virus 3 hypothetical protein 1 (YP_009337434.1)	50.80%	55%	0
Apple tombus-like virus 2	6R	914.6	4340	Cowpea tombusvirid 1 RdRp (APA23091.1)	66.70%	65%	0.00E+00
Apple virus A	3R	18.32	5751	Rhizoctonia solani putative virus 1 hypthetical protein (QDW81310)	98.00%	51%	0.00E+00
Apple virus B	5R	41.19	9234	Penicillium glabrum negative-stranded RNA virus 1 RdRp (QDB75014)	35.00%	30%	3.00E-111
Apple virus C	1R, 3R	73.9	4242	Hubei narna-like virus 8 RdRp (APG77202.1)	38.10%	41%	8.00E-106
Apple virus D	2R	26.87	7966	Riboviria sp RdRp (QDH87729)	33.00%	28%	1.00E-75

a. Names for the putative novel viruses were determined based on the BLASTx analysis.

**Fig 1:**
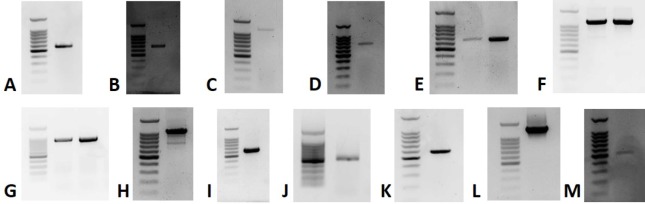
Detection of novel viruses by RT-PCR. Detection of A) Apple ilarvirus 1 in 5R, B) Apple narna-like virus 2 in 3R, C) Apple ourmia-like virus 1 in 3L, D) Apple ourmia-like virus 2 in 5R, E) Apple ourmia-like virus 3 in 3R and 6R, F) Apple partiti-like virus 1 in 1R and 2R, G) Apple partiti-like virus 2 in 1R and 2R, H) Apple partiti-like virus 3 in 2R, I) Apple tombus-like virus 2 in 6R, J) Apple virus A in 3R, K) Apple virus B in 5R, L) Apple virus C in 1R, and M) Apple virus D in 2R. Sizes for the ladder are, in descending order, 1.5 kb, 1 kb, 900 bp, 800 bp, 700 bp, 600 bp, 500 bp, 400 bp, 300 bp, 200 bp, and 100 bp.

To confirm the presence of the putative novel viruses, RT-PCR was used to detect the viruses. Thirteen of the novel viruses were amplified, generating the expected band (Fig 1, S2 Table). For the remaining four, inability to detect may indicate that they are present at titers below the threshold required for detection by RT-PCR.

**Fig 2:**
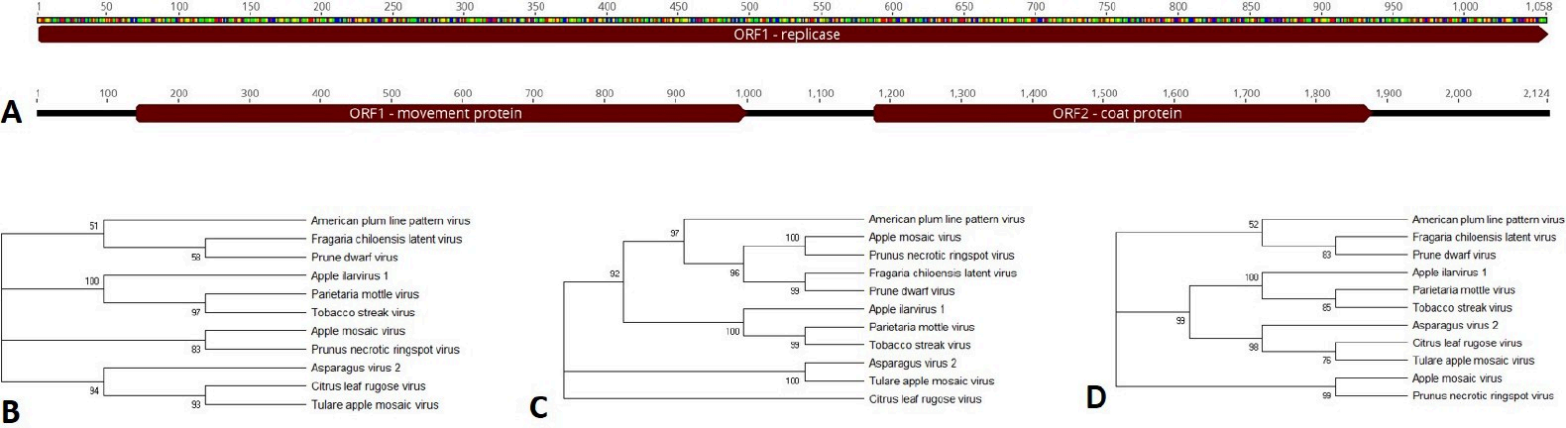
Apple ilarvirus 1. A) RNA2 and RNA3 for Apple ilarvirus 1. The predicted open reading frames for the replicase in RNA2 and the coat and movement proteins in RNA 3 are shown. B) The protein phylogenetic tree for the replicase, C) movement protein, and D) coat protein. Accession numbers for nucleotide and protein sequences are provided in S4 Table.

**Fig 3:**
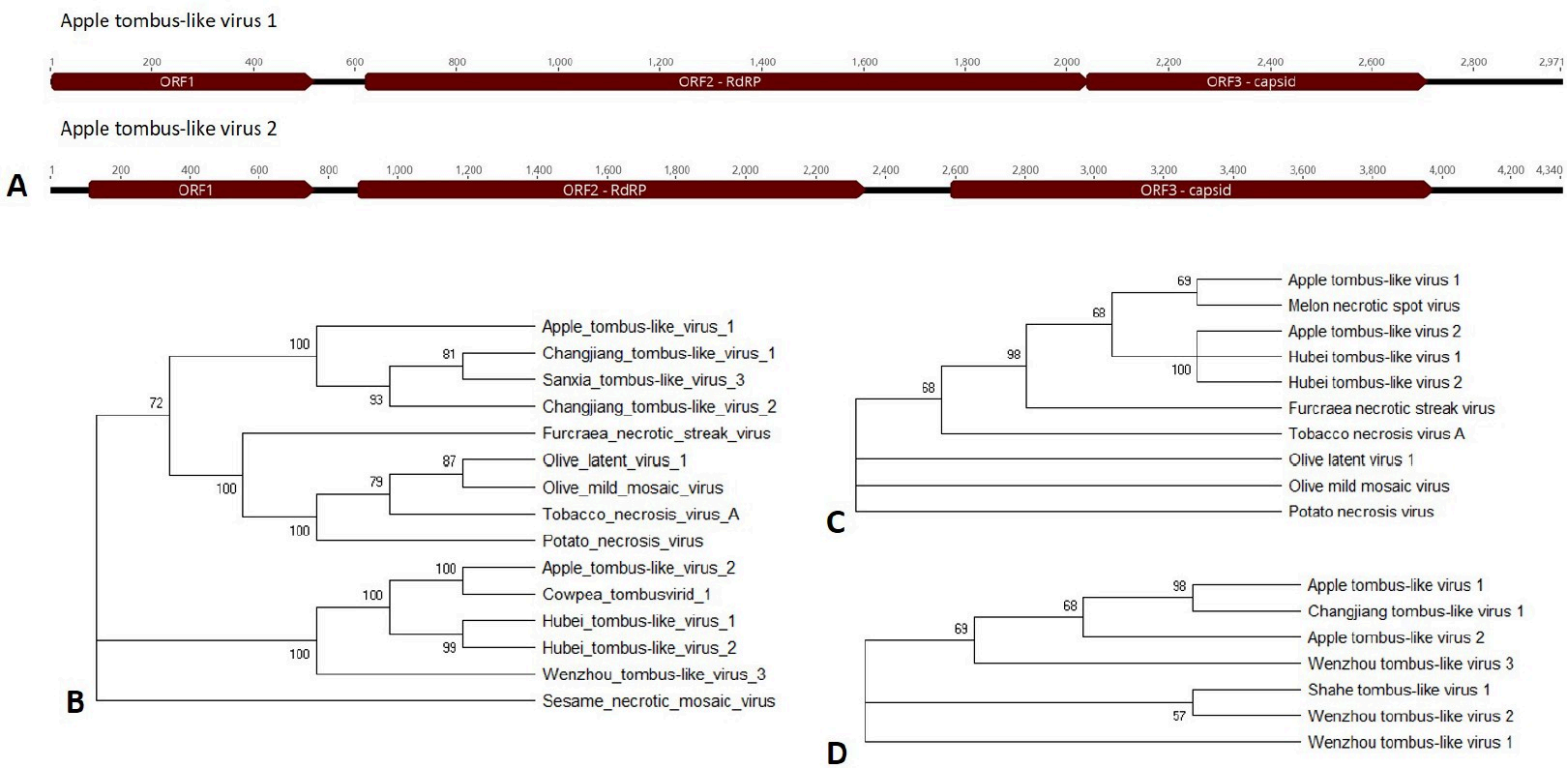
Apple tombus-like viruses 1 and 2. A) Partial genomes showing the open reading frames for ORF1, RdRp, and the capsid. B) Phylogenetic tree for the RdRp amino acid sequence, C) ORF1, and D) the capsid. Accession numbers are provided in S4 Table.

**Fig 4:**
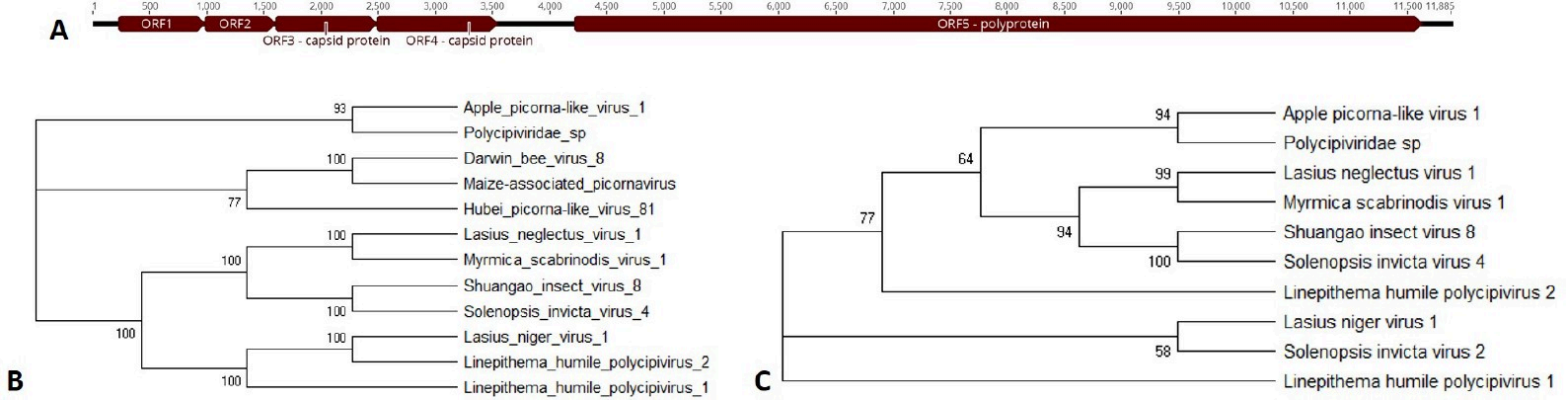
Apple picorna-like virus 1. A) Genome showing five open reading frames, including two capsid proteins and a polyprotein. B) Phylogenetic tree of the polyprotein amino acid sequence. C) Phylogenetic tree of the capsid (ORF4) protein. Accession numbers are provided in S4 Table.

**Fig 5:**
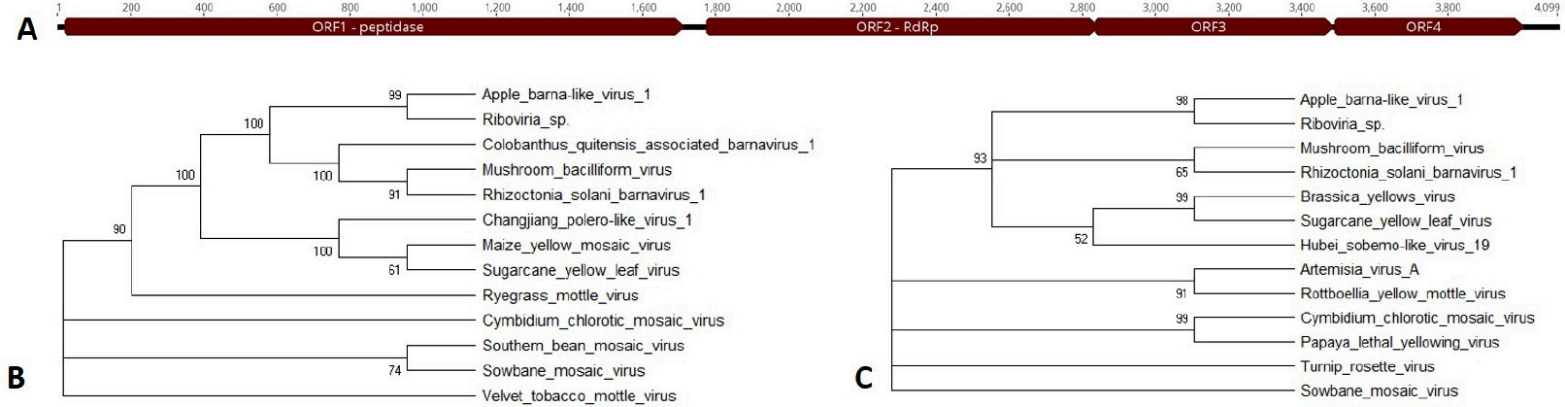
Apple barna-like virus 1. A) Genome showing four open reading frames, including a peptidase and RdRp. B) Phylogenetic tree of the RdRp and C) the peptidase. Accession numbers are provided in S4 Table.

**Fig 6:**
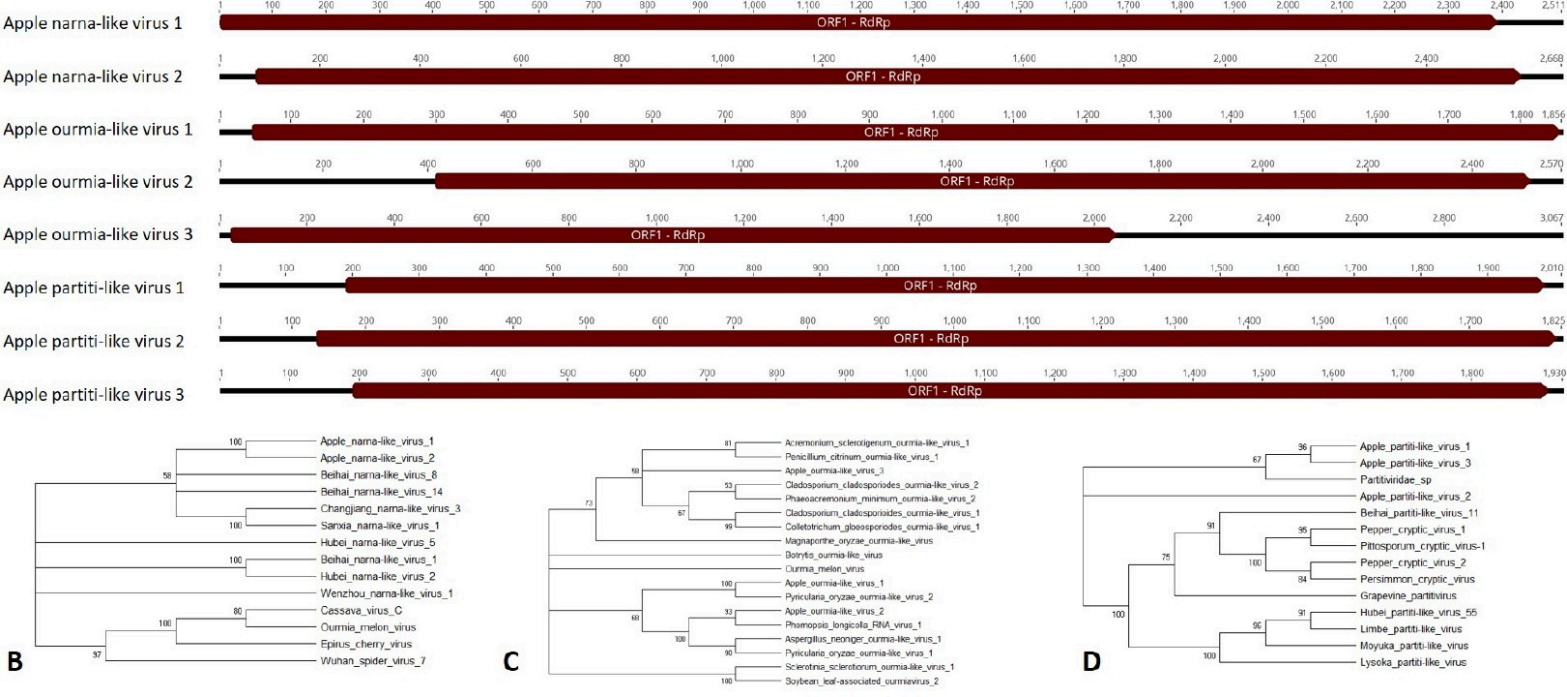
Apple narna-like, ourmia-like, and partiti-like viruses. A) Genomes showing the RdRp for Apple narna-like viruses 1 and 2, Apple ourmia-like viruses 1, 2, and 3, and Apple partiti-like viruses 1, 2, and 3. B) Phylogenetic tree of the RdRp for Apple narna-like viruses 1 and 2. C) Phylogenetic tree of the RdRp for Apple ourmia-like viruses 1, 2, and 3. D) Phylogenetic tree of the RdRp of Apple partiti-like viruses 1, 2, and 3. Accession numbers are provided in S4 Table.

**Fig 7:**
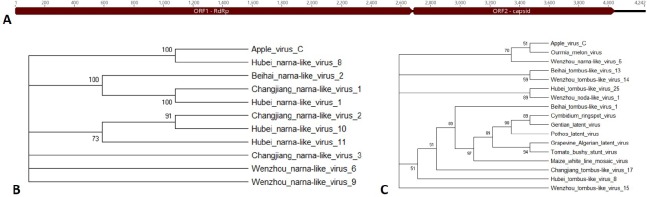
Apple virus C. A) Genome of Apple virus C showing predicted open reading frames for the RdRp and capsid. B) Phylogenetic tree of the RdRp. C) Phylogenetic tree of the capsid. Accession numbers are provided in S4 Table.

**Fig 8:**
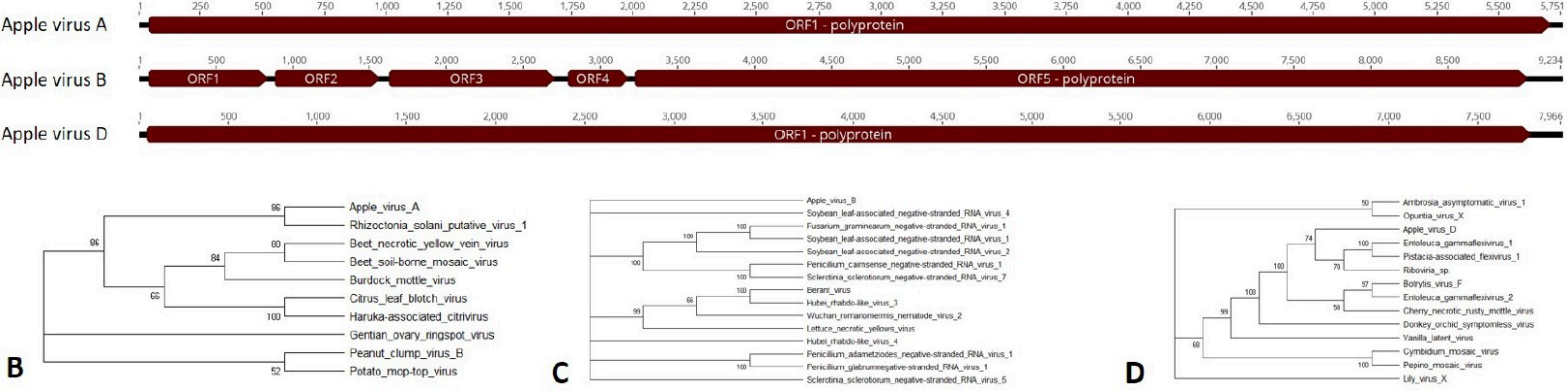
**Apple viruses A, B, and D.** A) Genomes showing predicted open reading frames for Apple virus A, B, and D. B) Phylogenetic tree of polyprotein for Apple virus A, C) Apple virus B, and D) Apple virus D. Accession numbers are provided in S4 Table.

Open reading frames for the novel viruses were predicted based on assembly and homology to other viral proteins in the protein database (Figs 2–8). Each of these viral genomes are incomplete, missing, at a minimum, the 5’ and 3’ UTRs. Nearly complete are the Apple barna-like virus 1 and Apple picorna-like virus 1, and Apple narna-like virus 2. For the ilarvirus, almost all of RNA3 was sequenced, while only part of RNA2 was sequenced and RNA1 was not detected. For the ourmia-like viruses, and the partiti-like viruses, only the RdRp was identified; for all six viruses the RdRp is complete, indicating that only the 5’ and 3’ ends are missing for those RNAs. For Apple narna-like virus 1, a partial RdRp was sequenced. Apple viruses A, B, C, and D are too different from any known viruses to estimate how complete their genomes are.

### Phylogenetic analysis

#### Apple ilarvirus 1

Two ilarviruses were detected among the six trees, ApMV and a putative novel ilarvirus, Apple ilarvirus 1. This novel virus was detected in the root tissue of tree 5, a Sugar Bee scion on an M9 rootstock (Tables 1 and 4). Ilarviruses have a tripartite genome [24], however only RNA2 and RNA3 were detected. RNA3 was sequenced nearly in full containing domains for both the movement protein and the capsid protein while RNA2, containing the replicase, was only partially sequenced (Fig 2A). RNA3 was present at a much greater incidence than RNA2 (an average read depth of 91 for RNA3 and 11 for RNA2; Table 4), which likely accounts for a greater percentage of RNA3 being sequenced than RNA2. Initial BLASTn searches indicated that both contigs are ilarvirus-like, with 72% and 71% nucleotide identity to Tobacco streak virus and Blackberry chlorotic ringspot virus, respectively. As ApMV, also an ilarvirus, was present in the samples, homology to this virus was investigated. Percent nucleotide identity with ApMV for RNA 2 and RNA3 were 48.7% and 48.4%, respectively, indicating that these RNAs represent a virus distinct from ApMV. From BLASTx, RNA2 and RNA3 were most similar to Blackberry chlorotic ringspot virus and Parietaria mottle virus, respectively (Table 4). Phylogenetic analysis was performed on the three predicted proteins in Apple ilarvirus 1. Analysis of the translated replicase, movement protein, and coat protein showed that Apple ilarvirus 1 was a sister taxon to Parietaria mottle virus and Tobacco streak virus (TSV) (Fig 2B–2D) with significant bootstrap support. Percent identities for the replicase, movement, and coat proteins when aligned with Parietaria mottle virus were 38.9%, 42%, and 40.5%, respectively. For TSV, they were 37.8%, 40.4%, and 32.3% respectively. Parietaria mottle virus and TSV belong to subgroup one of the ilarvirus family [24].

#### Apple tombus-like viruses 1 and 2

Apple tombus-like virus 1 was found in the roots of a symptomatic Firestorm scion on G.935 rootstock and on asymptomatic Sugar Bee on M9, while Apple tombus-like virus 2 was found in the roots of a symptomatic Honeycrisp on G.935 (Tables 1 and 4). Three open reading frames were predicted for the Apple tombus-like virus 1 and Apple tombus-like virus 2 genomes: RdRp, ORF1, and the capsid (Fig 3A). These viruses are distinct from each other, sharing only 43.3% nucleotide identity. The movement protein was missing from both suggesting that these genomes may be incomplete. Apple tombus-like virus 1 only had a sequencing read depth of 36.81, but for Apple tombus-like virus 2 it was much higher at a depth of 914.6 (Table 4). The BLASTx top hits were to tombus-like viruses: Sanxia tombus-like virus 3 for Apple tombus-like virus 1 and Cowpea tombusvirid 1 (a newly discovered virus from cowpea) [25] for Apple tombus-like virus 2 (Table 4). Phylogenetic analysis of the amino acid sequences for the three identified proteins also supported that these two viruses are tombus-like. For the RdRP, Apple tombus-like virus 1 clustered with Changjiang tombus-like viruses 1 and 2 and Sanxia tombus-like virus 3, with percent identities of 57.3%, 56.3%, and 54.9%, respectively, while Apple tombus-like virus 2 clustered with the Cowpea tombusvirid 1 with a percent identity of 65.2% (Fig 3B). For both clusters, the bootstrap value was significant. For ORF1, Apple tombus-like virus 1 clustered weakly with Melon necrotic spot virus (percent identity of 25.3%) and Apple tombus-like virus 2 significantly with Hubei tombus-like viruses 1 and 2 (percent identity of 24.4% and 27.3%, respectively) (Fig 3C). For the capsid, Apple tombus-like virus 1 clustered significantly with Changjiang tombus-like virus 1 (percent identity of 27%) while Apple-tombus like virus 2 clustered weakly with this same virus (percent identity of 18.8%) (Fig 3D).

#### Apple picorna-like virus 1

Apple picorna-like virus 1 was detected in the leaf tissue of the healthy G.935 tree and de novo assembly produced a contig of over 11.8 kb in length with five predicted open reading frames (Fig 4A). While a BLASTn search of the whole contig revealed no nucleotide similarity to any extant viral species, a BLASTx search revealed limited homology to Polycipiviridae (Table 4). This was also reflected in the phylogenetic trees for the RdRp and one of the capsid proteins (Fig 4B and 4C). In both instances, Apple picorna-like virus 1 clustered significantly with Polycipiviridae sp., and most closely to an unnamed virus isolated from *Pteropus lylei* stool. The percent identity for the alignment of the RdRp and capsid with this unnamed virus were 24.2% and 24.3% respectively. Polycipiviridae species are typically found in ants, with Apple picorna-like virus 1 and the unnamed Polycipiviridae virus in fruit bat stool [26] as exceptions.

#### Apple barna-like virus 1

Like Apple picorna-like virus 1, Apple barna-like virus 1 was also found in the leaf tissue of a healthy G.935 tree (Table 4). Four open reading frames, including the peptidase and RdRp were predicted for Apple barna-like virus 1 (Fig 5A). The BLASTn and BLASTx top hits for Apple barna-like virus 1 were a Riboviria sp. isolated from soil (Table 4). In performing phylogenetic analysis on the amino acid sequences of the peptidase and RdRp, it was found that both clustered with the Riboviria sp. soil isolate (Fig 5B and 5C). Percent identities for the RdRp and peptidase, when aligned to the Ribiovira sp. soil isolate, were 48.2% and 30.8%, respectively. Together, these two viruses cluster with fungal barnaviruses making it likely that this novel virus is also a barnavirus. However, the genomic structure does differ from typical barnaviruses. There are two ORFs, not one, following the RdRp and the ORF1 that typically overlaps the 5’ end of the peptidase is missing (Fig 5A) [27]. For this reason the virus has been termed “barna-like.”

#### Apple narna-like virus 1 and 2

In the roots of a Firestorm on G.935, two contigs resembling narna-like viruses were detected (Table 4, Fig 6A). Narnaviruses are monopartite with genomes ranging from 2.5 to 2.9 kb in length and possess only an RdRp [28]. Based on this information, it is likely that all of the genome but the very ends of these two narna-like viruses were assembled. While both these narna-like viruses had amino acid sequence similarity to Wenzhou narna-like virus 1 (Table 4) and they clustered together significantly in the phylogenetic tree (Fig 6B), they are distinct from each other. By nucleotide alignment they share with each other only 49.7% identity and by amino acid alignment of the RdRps they share 32.9% identity. Apple narna-like virus 1 and Apple narna-like virus 2 clustered together significantly and weakly clustered with the Behai narna-like viruses 8 and 14, Changjiang narna-like virus 3, and Sanxia narna-like virus 1 (Fig 6B). Percent identities for Apple narna-like virus 1 with these four viruses were 22.6%, 22.6%, 22.4%, and 21.8% respectively and for Apple narna-like virus 2 was 22.6%, 15.2%, 21.1% and 18.4% respectively.

#### Apple ourmia-like viruses 1, 2, and 3

Three contigs with homology to ourmia-like viruses were identified (Fig 6A, Table 4). Apple ourmia-like virus 1 was found in the leaf tissue of a Firestorm on G.935 (Table 4). The other two were found in the roots: Apple ourmia-like virus 2 was detected in the roots of a Sugar Bee on M9 and Apple ourmia-like virus 3 was found in both a Firestorm on G.935 and a Honeycrisp on G.935 (Table 4). Ourmiaviruses are tripartite, with the RdRp, movement protein, and coat protein each encoded on separate RNAs [29]. Only the RdRp containing segment was identified for these three viruses–the remaining segments may have been too different in sequence from any known viruses to be identified by BLASTx analysis. The RdRp amino acid sequence was used for phylogenetic analysis. Apple ourmia-like virus 1 significantly clustered with Pyricularia oryzae ourmia-like virus 2 (50% amino acid identity) and Apple ourmia-like virus 2 significantly with Phomopsis longicolla RNA virus 1 (50.1% amino acid identity). Apple ourmia-like virus 3 was associated with two clusters of fungal ourmia-like viruses (Fig 6C). This last virus had no hits in the BLASTn search and had only 33% identity with Cladosporium cladosporioides ourmia-like virus 1, the top hit in the BLASTx search (Table 4).

#### Apple partiti-like viruses 1, 2, and 3

Three partiti-like viruses were detected in the root tissue of the Firestorm/G.935 trees that were symptomatic: Apple partiti-like viruses 1 and 2 were detected in all three trees and Apple partiti-like virus 3 was detected in trees 2 and 3 (Table 4). Partitiviruses are bipartite [30], yet for all three viruses detected in these trees only the segment containing RdRp was detected (Fig 6A). BLASTx searches indicated that all three had some homology to recently discovered Partitiviridae sp. found in soil (Table 4). In phylogenetic analysis of the RdRp sequences, Apple partiti-like viruses 1 and 3 clustered together significantly and weakly with a Partitiviridae sp. (Fig 6D). Apple partiti-like virus 2 did not significantly cluster with any partitiviruses (Fig 6D). Apple partiti-like viruses 1 and 3 were distinct from each other, sharing 35.5% identity for the RdRp amino acid sequence. All three viruses had very high sequence coverage, ranging from an average depth of 234 to 783 reads.

#### Apple virus C

Apple virus C was detected in the roots of Firestorm/G.935 trees 1 and 3 (Table 4). For Apple virus C, two open reading frames encoding the RdRp and capsid were predicted (Fig 7A). Phylogenetic trees were constructed for both proteins (Fig 7B and 7C). The RdRp clustered significantly with Hubei narna-like virus 8 and had a percent identity of 37.7% (Fig 7B). However, the capsid clustered weakly with Ourmia melon virus and had a percent identity of only 11% (Fig 7C). The presence of a gene encoding a capsid protein on the same RNA as the RdRp sets it apart from both narnaviruses and ourmiaviruses [28, 29], making it difficult to classify this virus.

#### Apple viruses A, B, and D

Apple viruses A, B, and D are too different from known viruses to be tentatively assigned to a specific family. Single open reading frames encoding a polyprotein were predicted for Apple virus A and Apple virus D (Fig 8A). For Apple virus B, five open reading frames were predicted with the fifth encoding a polyprotein (Fig 8A). Apple virus A had a BLASTx hit to a Rhizoctonia solani putative virus 1 (Table 4). The RdRp of Apple virus A clustered strongly with this virus and had a percent identity of 50.4% (Fig 8B). Apple virus B could not be grouped phylogenetically (Fig 8C), however in BLASTx analysis it was most similar to Penicillium glabrum negative-stranded RNA virus 1 (Table 4). Alignment of the RdRp amino acid sequences for these two viruses revealed a percent identity of 24.8%. Apple virus D had some similarity to Riboviria sp. based on a BLASTx search (Table 4) and a phylogenetic tree of the polyprotein shows it clustering with a taxon containing Entoleuca gammaflexivirus 1, Pistacia-associated flexivirus 1 and Riboviria sp. (Fig 8D). The percent identity for the RdRp for the three viruses was 22.5%, 23.9%, and 26.7%, respectively. A coat protein was not detected for this virus.

## Discussion

The diagnosis of new and emerging diseases of plants has been greatly aided by the development of technologies such as high-throughput sequencing [5]. Yet, our ability to discover viruses has been outpacing our ability to associate them with a particular disease, either by themselves, or synergistically with other viruses in mixture. This is particularly true of viruses in perennials, which by their very nature are long-lived and stationary, providing ideal conditions for infection by one or more viruses over time [31]. In this study we began by examining a disease, apple decline, and through applying an HTS approach we found far more known and putative viruses than expected. This raises the question facing plant pathologists today: how do we translate this data into beginning to understand the cause of a disease?

### Known viruses

In the absence of a smoking gun we must proceed by analogy and review what is known about the viruses that are present. Here we found a total of eight previously described viruses and one viroid. Of these, ACLSV, ApMV, ASGV, and ASPV are common in apples in Washington state (Table 3). These are known to frequently occur together [32, 33] and, although they are mostly symptomless when present in commercial cultivars they have been reported to induce symptoms in specific apple cultivars [32–35]. ASGV so far is only known to be symptomatic on Virginia Crab (*Malus sylvestris)*, where it causes grooving in the stems [35]. This virus can cause necrosis in the graft union and early dropping of leaves [36]. ACLSV can also cause decline and death of trees, but only on specific rootstocks, particularly Asami [37]. ACLSV infection in trees on this rootstock can cause a hypersensitive reaction at the graft union and failure of the trees, which is known as topworking disease [37]. ASPV is known to cause stem pitting in Virginia crab and in apple cultivars Charden and Reinette Clochard, but is often asymptomatic in commercial cultivars [38]. ApMV infection can result in yellow to cream colored spots on leaves, leading to premature drop of symptomatic leaves [39]. None of these four viruses has been consistently associated with the decline currently observed in Washington state as they are observed in both healthy and declining trees (Table 3). If they do play a role in decline of these trees, it is unlikely that they are the single responsible factor.

The frequency of recently discovered AhVd [19], ARWaV1 and 2 [15] and CCGaV [16] in apples in Washington state is not known, however they were all detected in at least one sample from this work; AhVd was present in all sequenced trees (Table 3). While CCGaV has been associated with concave gum disease in citrus [16], it has also been reported in apples [40]. Currently, the virus has not been associated with any symptoms or diseases in apple. ARWaV1 and 2 have been associated with apple rubbery wood disease [15], however the symptoms of that disease are distinct from what is observed in apple decline. AhVd has been observed in Pacific Gala trees that exhibit limb swelling and cracking [19], however those symptoms also differ from apple decline. Lastly, AGCaV was detected in both healthy and declining plants (Table 3). This virus was discovered in trees exhibiting symptoms of apple green crinkle, which can cause decline [41]. Like the more common viruses, the presence of AhVd and these four viruses in both healthy and declining plants indicates that if they do play a role in the disease, they are not responsible for the disease alone, but may be part of a larger group of factors that contribute to this disease. In addition ALV1, which has been detected in both symptomatic and asymptomatic trees in the eastern United States [2], was not observed in any of the six trees examined here.

### Novel apple viruses

Traditionally, the most understood plant viruses are those that caused disease on a given host species, and/or on herbaceous or woody biological indicators [42]. But, there are many instances where these ‘known’ viruses cause no disease. This may, primarily, be due to host physiological or genetic differences where these viruses lacked the right effectors to induce disease [5]. Yet, there has always remained the question of whether a factor, such as another virus acting synergistically, was missing.

The cause of apple decline has remained elusive in both the eastern United States and in Washington state. A study by Singh et al. [4] ruled out the involvement of ASGV, ASPV, and ACLSV, while in this study we have found several more known viruses that require investigation. But what else? To investigate the possibility that an as yet unknown virus may be involved, non-apple contigs without any hits using BLASTn (megablast) were subjected to BLASTx analysis. This revealed an unexpected diversity of putative viral sequences. A total of seventeen putative novel viruses were identified, thirteen of which could be tentatively assigned to seven known viral families (Table 4). Thirteen of the viruses were validated by RT-PCR (Fig 1). The remaining four, Apple barna-like virus 1, Apple narna-like virus 1, Apple picorna-like virus 1, and Apple tombus-like virus 1, may have been at too low a titer to be detected by endpoint RT-PCR.

Some of these novel viruses seem to be plant-infecting viruses. These include Apple ilarvirus 1, Apple tombus-like viruses 1 and 2, Apple partiti-like viruses 1, 2, and 3, and Apple picorna-like virus 1. Ilarviruses infect plants [24] and the RNA2 and RNA3 of Apple ilarvirus 1 are most homologous to known plant viruses (Table 4). Similarly, tombusviruses also infect plants [43]. Apple tombus-like virus 2 in particular was present at a very high titer: the average read-depth was 914.6, higher than any of the other novel viruses (Table 4). The Apple partiti-like viruses 1, 2, and 3 were also sequenced at high read depths, ranging from 234 to 783 (Table 4). Partitiviruses are known to infect both fungi and plants [44] raising the possibility that these are plant viruses, however additional work is needed to determine if apple is their host. Most interesting was Apple picorna-virus 1, which was most closely related to the Polycipiviridae genus (Table 4). Polycipiviridae species are known to infect ants [45]. The genome in size and structure is similar to known species in this genus, with four open reading frames followed by an intergenic region and a polyprotein [45]. The Polycipiviridae species to which the RdRp had the closest homology was identified in the stool of a fruit bat, *Pteropus lylei* [26]. Apple picorna-like virus 1 and the virus found in the *Pteropus lylei* stool are the only two instances so far of viruses with homology to members of the Polycipiviridae genus identified outside of ants.

Several of the viruses appeared to be infecting associated fungal species. These include Apple barna-like virus 1, Apple narna-like viruses 1 and 2, and Apple ourmia-like viruses 1, 2, and 3. For all six of these viruses, phylogenetic analysis indicated that they were related to known fungal infecting viruses (Figs 5 and 6). These were likely sequenced along with fungi that were associated with the plant tissues. The remaining four viruses cannot be assigned to families and therefore it is uncertain if they were infecting the sequenced trees themselves or potential associated fungal species. Of these four, Apple virus D is most likely infecting an associated fungal species as its RdRp was most closely associated with known fungal viruses (Fig 8D). Apple virus C had some similarity to both narnaviruses and ourmiaviruses. Some data have indicated that the ourmiaviruses arose from the combination of genomic segments from different viruses [46]. Given the number of viruses that can be present in a single organism, it is possible that Apple virus C also arose from rearrangements among multiple viruses.

While all the novel viruses appeared to be missing at the very least the 5’ and 3’ UTRs, some were missing whole genes. For the multipartite viruses, RNAs encoding capsid and movement proteins were not detected for the ourmia-like viruses, the RNA encoding the capsid for the partiti-like viruses, and the RNA1 encoding the RdRp for the Apple ilarvirus 1 were not sequenced. It could be that they were not sequenced or not identified as viral by BLASTn or BLASTx analysis. For the two tombus-like viruses, a movement protein was not detected, which characteristically is present on the single RNA that comprises the tombusvirus genome [43]. If the RNA1 for Apple ilarvirus and the movement protein for the tombus-like viruses are missing, and not just overlooked in the sequencing analysis, then this raises the question: how are these viruses able to function within the plant? It has been well documented that some viruses cannot function independently within a host and instead require helper viruses to provide the missing genes they need to function [47]. For example, helper viruses can complement dependent viruses lacking a functional movement protein. Transgenic plants expressing the Tobacco mosaic virus (TMV) movement protein were able to complement movement defective Red clover necrotic mosaic virus (RCNMV), allowing cell to cell movement of the virus. In the opposite situation in which TMV was defective and the plant transgenic for the RCNMV movement protein, cell to cell movement was also observed [48]. In another example, a strain of Cucumber mosaic virus defective for long-distance movement was able to spread throughout the plant when co-infecting with the helper virus Zucchini yellow mosaic virus strain A [49]. Given these examples, it is possible that Apple tombus-like viruses 1 and 2 are dependent viruses that are reliant upon the presence of a helper virus for movement. Apple ilarvirus 1 may also be a dependent virus, requiring a replicase from another virus to complement the missing RNA1. There is precedence for one or more the RNAs of a novel ilarvirus to go undetected in a sample. In a study of ilarviruses infecting *Prunus* species in Australia, two novel RNA2 sequences were detected, but no novel RNA1 or RNA3 sequences were identified [50]. In some of the trees in which the novel RNA2 sequences were identified, no other ilarvirus was found [50], in contrast to what is observed here, where the novel ilarvirus is co-infecting with another ilarvirus, ApMV. ApMV may be able to provide Apple ilarvirus 1 the missing replicase function. Lastly, given the number of viruses present in these samples, there is the possibility for a synergistic effect that could contribute to disease. Co-infection of Soybean mosaic virus and Bean pod mottle virus in soybean produce more severe symptoms than infection of either virus alone [51]. Similarly, Sweet potato feathery mottle virus and Sweet potato chlorotic stunt virus individually cause mild symptoms in sweet potato but co-infection results in the development of severe symptoms [52]. The number of viruses detected in the trees in this study range from five to fifteen. It is unknown if any of these viruses have a synergistic effect or if they may be contributing to apple decline.

### Tropism

For three of the trees included in this study, both root and leaf tissue was sequenced. Interestingly, for these three trees, many of the novel viruses that were detected in the roots were not detected in the leaf tissue. Viruses are not evenly distributed throughout a host and may show accumulation bias or adaptation for certain tissue types [53, 54]. For example, Pineapple mealybug wilt-associated virus-2 was found at much higher concentrations in the roots than in the leaves [55]. In extreme cases, such as with Citrus leprosis virus, the viruses remain localized in the cells they were introduced into by a vector and are unable to move systemically [56]. In this study, the frequency of viruses in the roots may be due to each species’ preferred tropism, or for the putative mycoviruses identified simply there due to the association between the fungal host or vector and the root system. An exception to this was Apple ourmia-like virus 1, which was detected in the leaf tissue (Table 4). It is also possible that some of the viral species may be accumulating in the roots though passive movement there through bulk flow from the vascular system, and are present in the upper parts of the plant at levels too low to detect [53].

What the frequency of viruses in the roots does highlight is the need to consider the entire host when studying plant viromes. Some virome studies have examined multiple tissues for their plant species of interest, either pooling samples or sequencing them separately [8, 10, 11, 14]. Czotter et al. [11] pooled extracts of shoot tips, young leaves, older leaves, tendrils and inflorescences of grapes for sequencing. Similarly, Jo et al. [10] pooled root, stem, leaf, and fruit tissue to sequence the pepper virome. For citrus plants, Matsumura et al. [14] sequenced leaf and root tissue separately. Roots and leaves were not always, but sometimes from the same plant [14]. Jo and Cho [8], in examining the lily virome, re-analyzed sequence data from previous studies that sequenced different tissue types. In examining different tissues, either separately or by pooling, these studies most likely provided a more complete picture of the viromes of the individual plants they examined than studies that sequenced only one tissue type.

## Conclusions

While next generation sequencing allows the detection of novel virus-like sequences, there are many questions that this technology cannot answer. Additional work will be needed to verify that these novel sequences represent viruses. The genomes of these viruses are incomplete and for those that are multi-partite, like the ormiaviruses and partitiviruses, obtaining full genomes will be a challenge. Although the hosts of these viruses, apple or fungal, are suspected, they cannot be confirmed with the existing data. How the viruses are transmitted is also unknown. Lastly, it is unknown if any of these viruses are pathogenic in any apple cultivar or if they have a synergistic effect when co-infecting. With 9 known viruses and viroids just in these trees and seventeen additional putative viruses, the number of combinations when addressing synergistic effects becomes very large (and even greater if multiple cultivars or rootstock/scion combinations are included).

Thus far the cause of apple decline has been elusive. Apple luteovirus 1 has been weakly associated with decline in the eastern United States [2], however it is unlikely that this virus is anything more than a contributing factor. The virus was not observed in the declining trees in this study. Like the ALV1 study, we also found that there was no one species, or set of species that were present in all disease expressing apple trees. However, our study encompassed a larger set of viruses. It is unknown how these viruses interact. Dieback, like that observed in apple decline, can have many causes. It may be that different combinations of viruses are responsible for the same outcome. These viruses may be interacting or they may be weakening the tree for another pathogen or stressor to cause decline. At this point there is no common thread distinguishing declining trees from healthy trees. A recent examination of a variety of factors, including weather, soil chemistry, viral infection, and microbial communities was conducted and no obvious difference was observed between symptomatic and asymptomatic trees [4]. This suggests that some other, as yet unidentified factor, is ultimately responsible for decline. Additional research will be required to address these questions and to determine what role, if any, these novel viruses have in apple decline.

## Supporting information

S1 Table Accession numbers for viral genomes. (XLSX)

S2 Table Number of reads mapped, percent of total reads, and genome coverage for all detected known viral genomes. (XLSX)

S3 Table Primer sequences and cycle condtions for detection of novel viruses. (XLSX)

S4 Table Accession numbers for sequences used in constructing phylogenetic trees. (XLSX)

S1 raw images Uncropped images of the gels presented in Fig 1. (PDF)
