# Supplementary material for: A bushel of viruses: Identification of seventeen novel putative viruses by RNA-seq in six apple trees
Source: PLoS One. 2020 Jan 13;15(1):e0227669. doi: 10.1371/journal.pone.0227669 (PMC6957168; doi:10.1371/journal.pone.0227669)
Supplement: S1 raw images — (PDF) [file pone.0227669.s005.pdf]

S1_raw_images

Below are the raw gel images for figure 1. The only modifications that occurred was cropping of images to exclude empty or unrelated (separate PCR run on the same gel) lanes or adjusting contrast of the whole image to make ladders and or bands more visible.

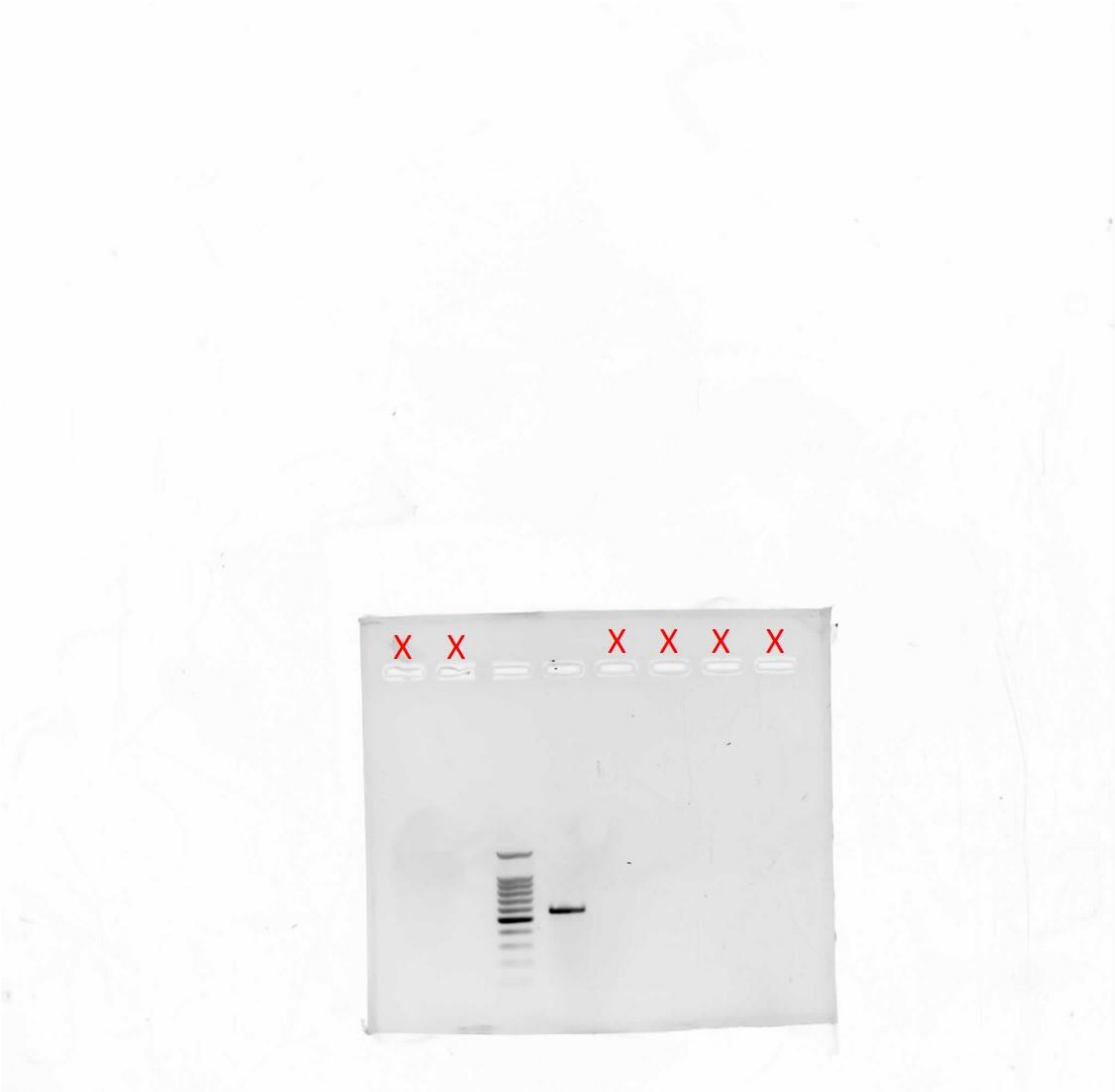

The above is the uncropped image for 1A. The image was cropped to the two lanes not marked with a red "X." The marked lanes were all empty except for the third which contained the no template control.

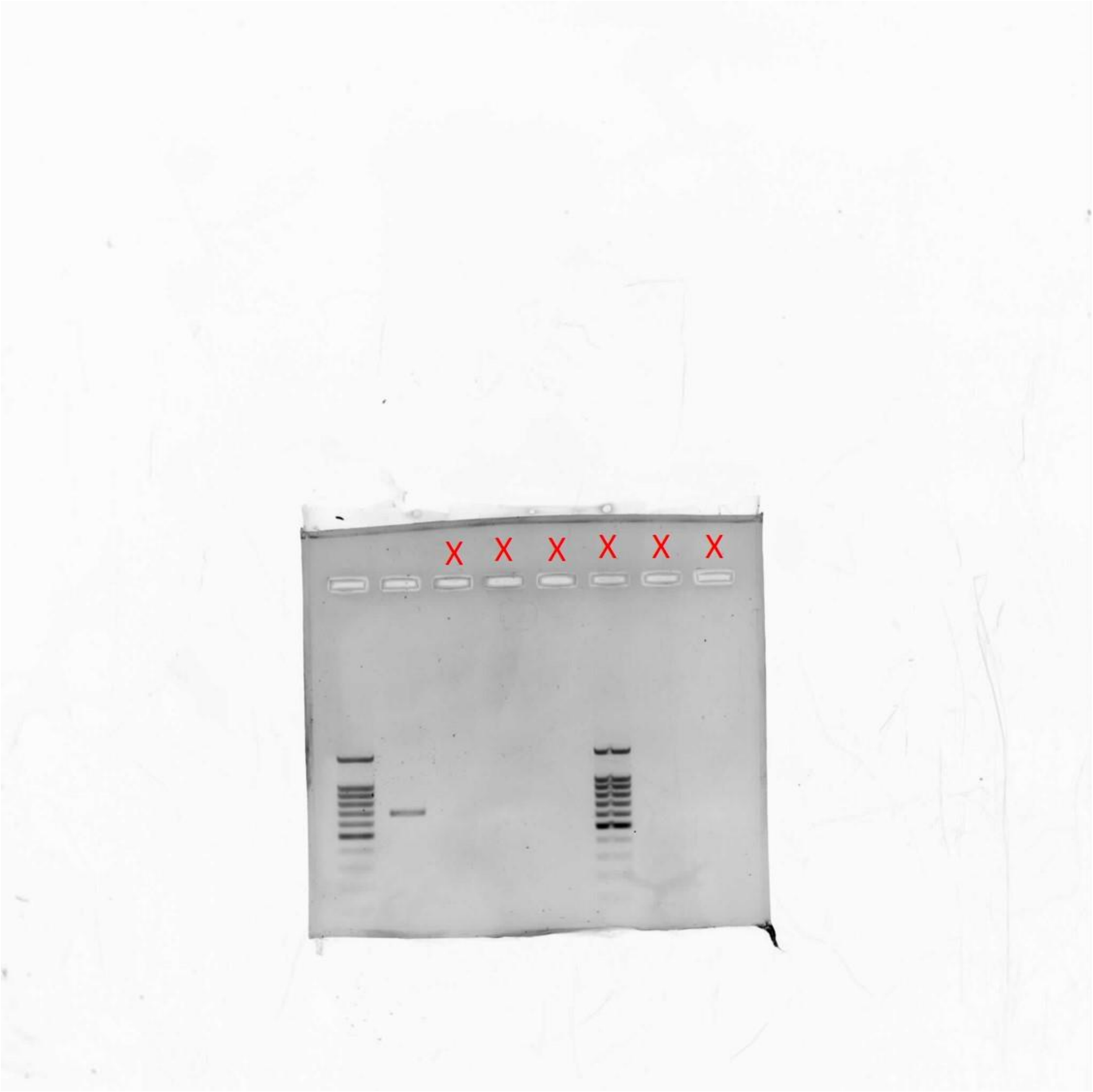

The above is the uncropped image for 1B. The rightmost 6 lanes were removed from the image by cropping. The first of these contained a no template control for the PCR in 1B, the second and third were empty, the fourth was ladder, and the fifth and sixth were a failed PCR unrelated to that in 1B.

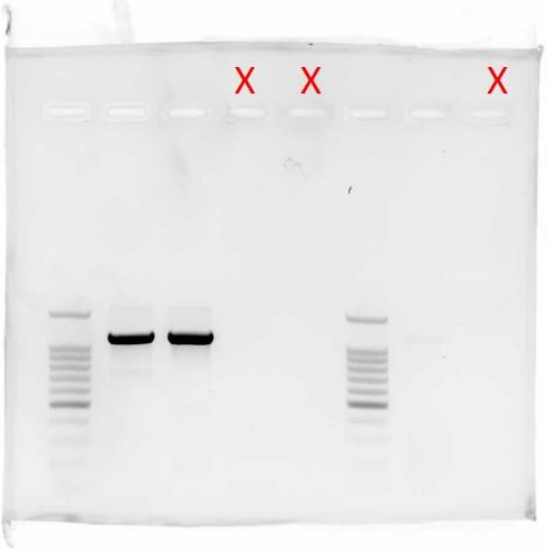

Above is the uncropped image for 1F and 1C. The first three lanes are 1F. Lane 4, marked with an X, is the no template control. Lane 5 is empty. Lanes 6 and 7 are 1C. The contrast was adjusted for 1C to make the band in lane 7 more visible (located between the top two most bands on the adjacent ladder in lane 6). Lane 8 was the no template control.

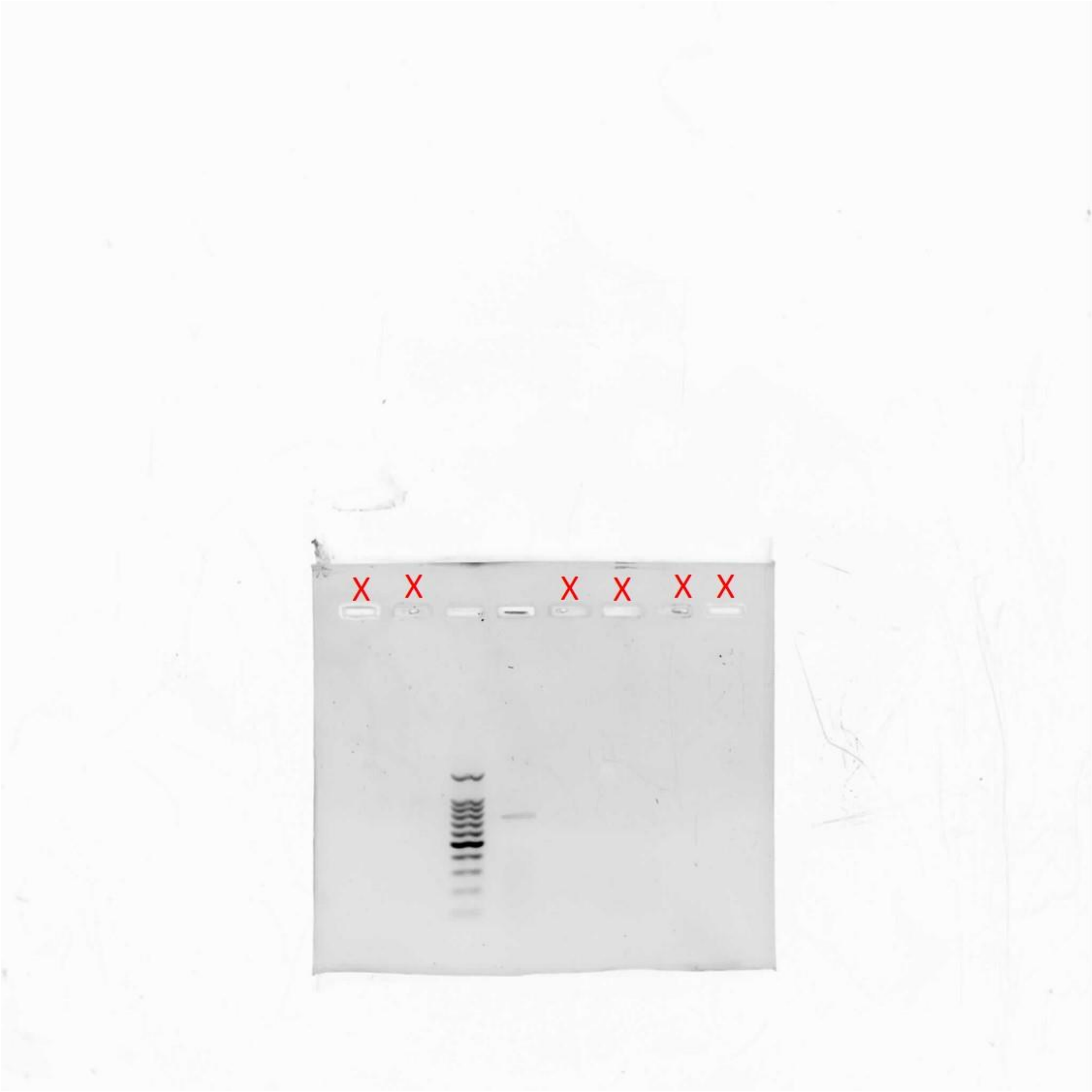

Above is the uncropped image for 1D. Contrast was also adjusted to improve visibility of the band in lane 4. Lanes 1, 2, and 6-8 were empty. Lane 5 contained the no template control.

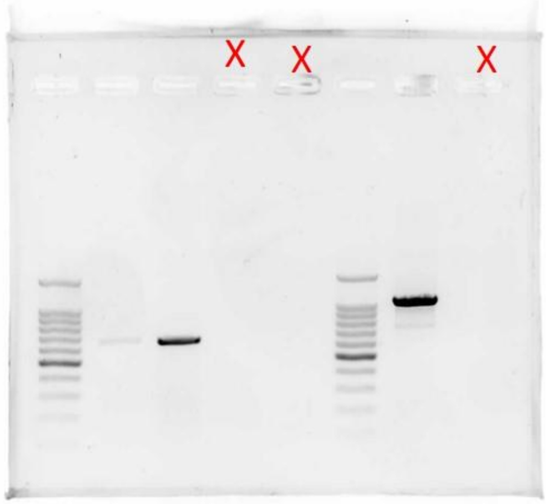

Above is the uncropped image for 1E and 1H. The contrast was adjusted slightly to improve visibility of the ladder and the band in land 2. Lanes 1-3 are 1E and lanes 6 and 7 are 1H. Laves 4 and 8 contain no template controls for each PCR. Lane 5 is empty.

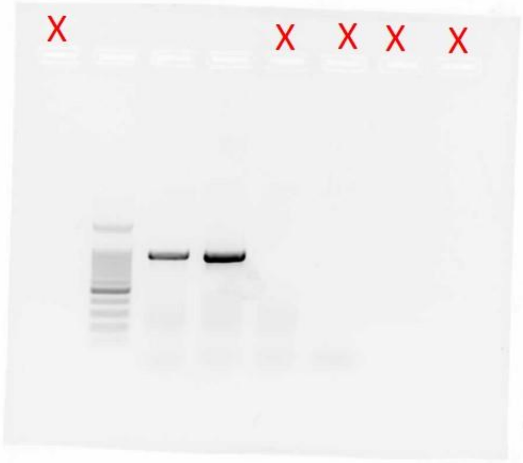

Above is the uncropped image for 1G. Lanes 1, 7, and 8 were empty. Lane 5 contained a failed PCR and lane 6 a no template control.

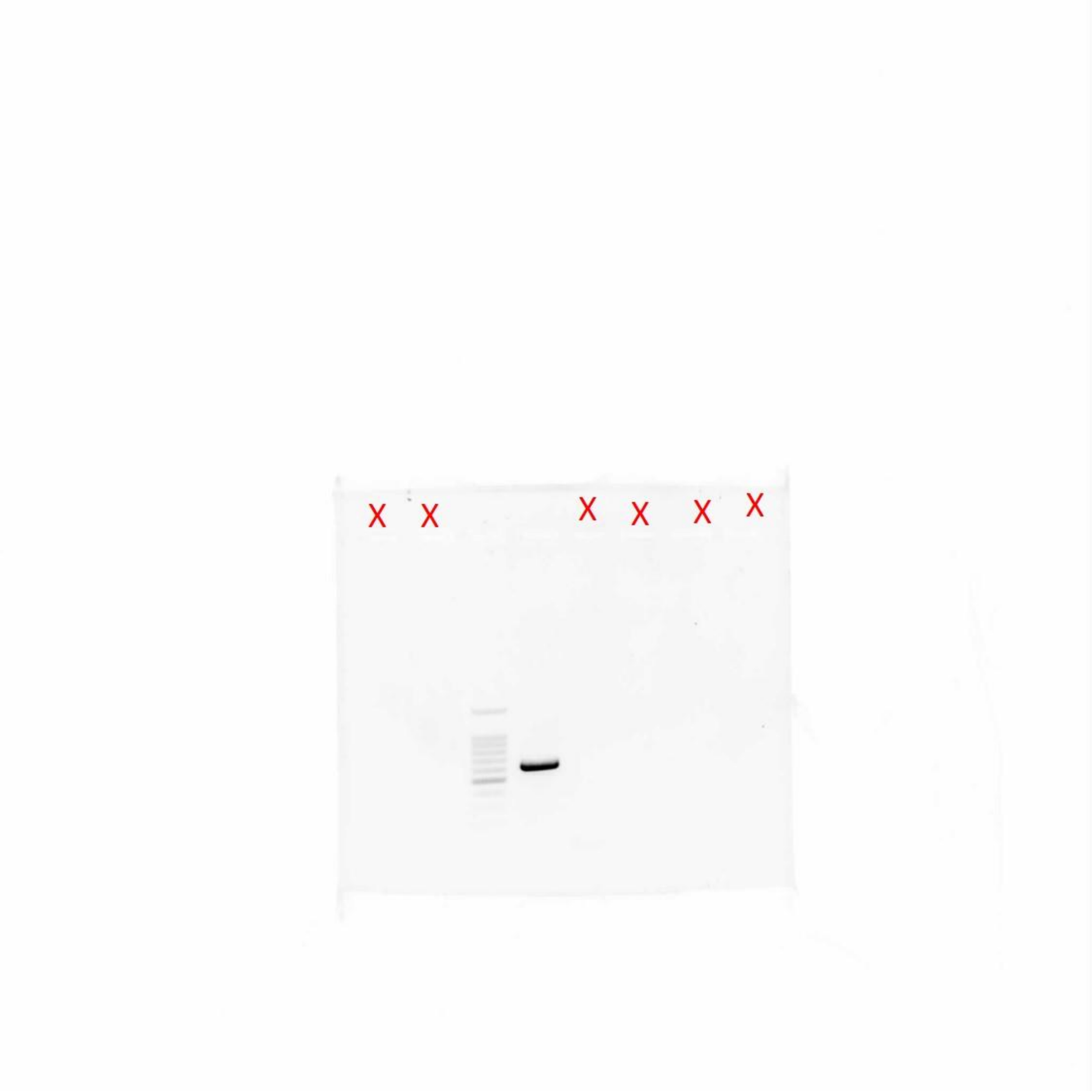

Above is the uncropped image for 1I. The contrast was adjusted for the paper to improve visibility of the ladder. All marked lanes were empty except for lane 5 which contained the no template control.

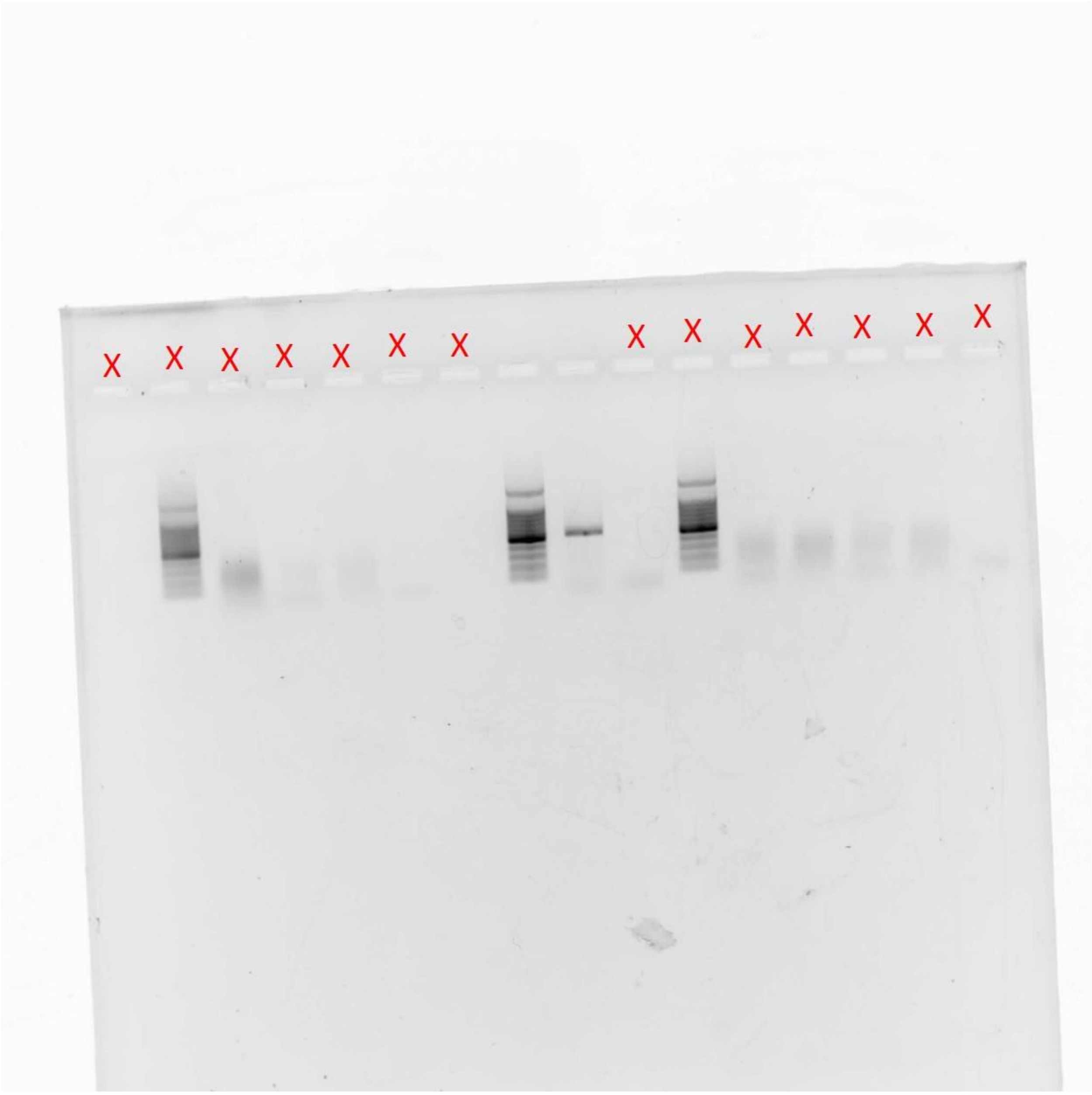

Above is the uncropped image for 1J. Lanes 1 and 7 are empty and lane 10 contains the no template control. The remaining lanes not included in 1J are failed PCRs.

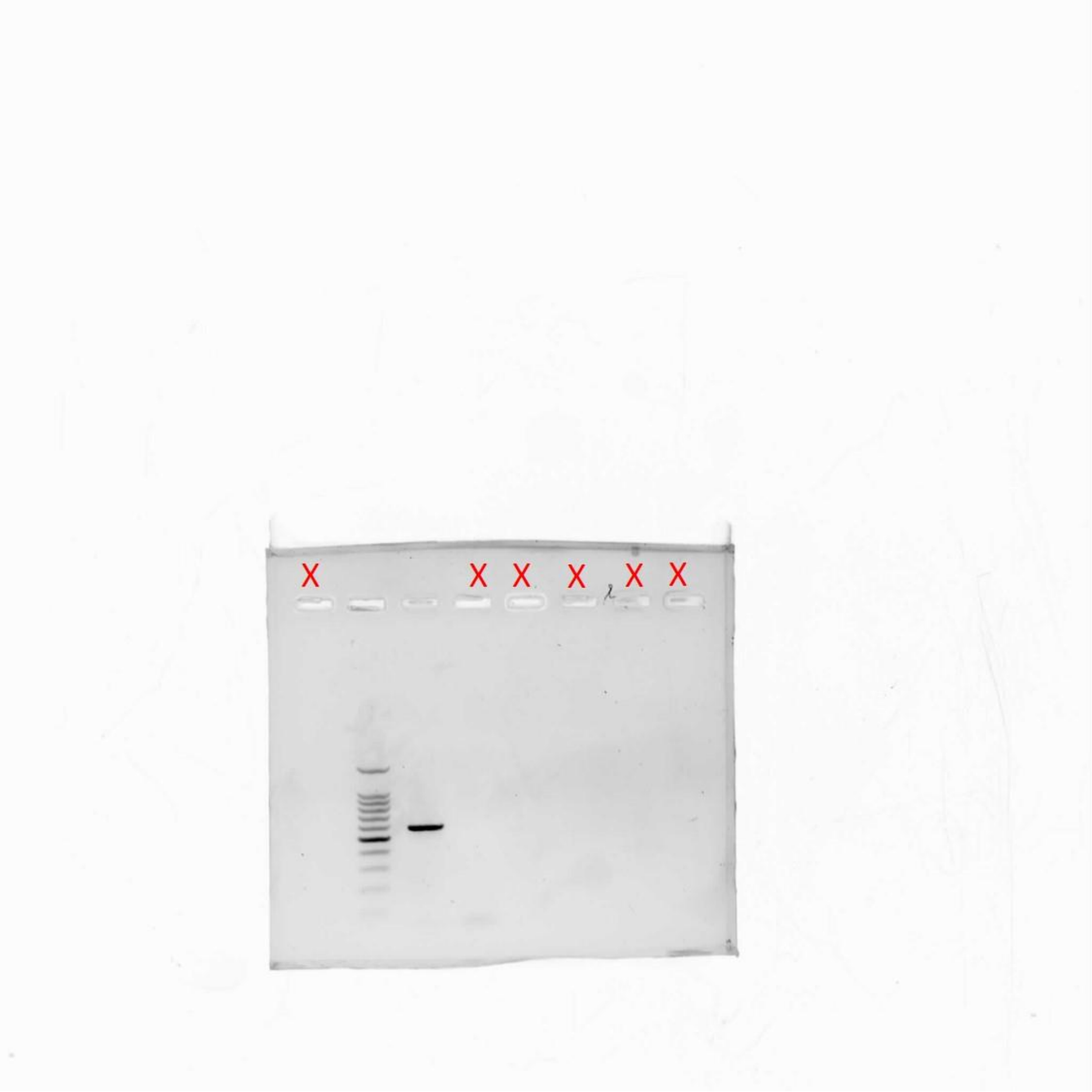

Above is the uncropped image for 1K. All marked lanes are empty except for lane 4 which contained the no template control.

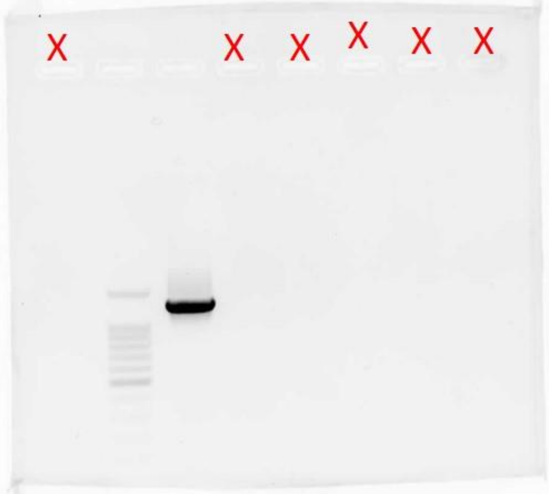

Above is the uncropped image for 1L. The contrast was adjusted slightly in 1L to improve visibility of the ladder. All marked lanes are empty except for lane 4 which contains the no template control.

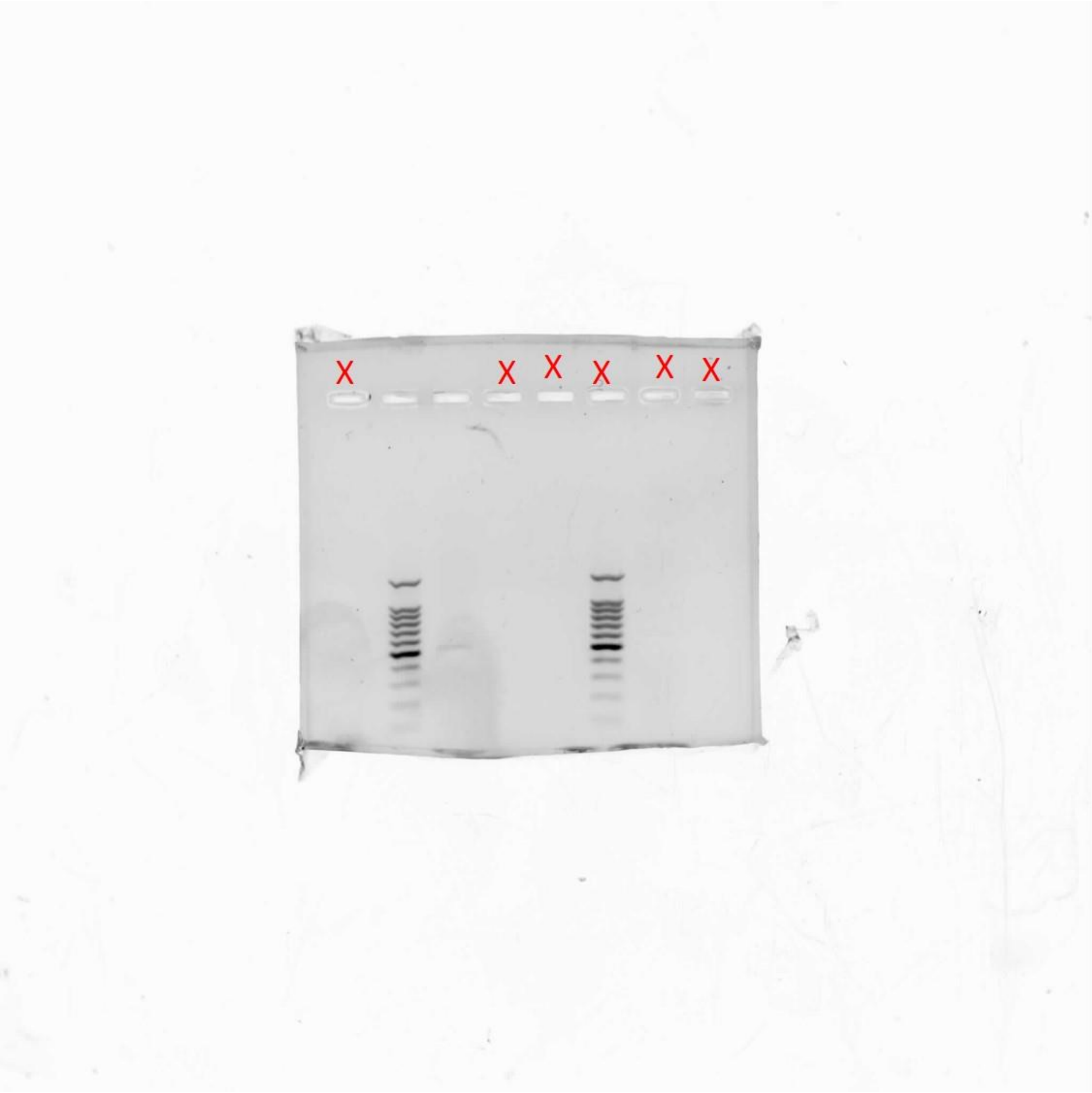

Above is the uncropped image of 1M. The contrast was adjusted slightly for 1M to improve visibility of the band in lane 3. Lanes 1 and 5 are empty. Lane 4 contains a no template control. Lanes 7 and 8 are a failed PCR.
